# Plasma activated medium prepared by a bipolar microsecond-pulsed atmospheric pressure plasma jet array induces mitochondria-mediated apoptosis in human cervical cancer cells

**DOI:** 10.1371/journal.pone.0272805

**Published:** 2022-08-08

**Authors:** Ara Jo, Hea Min Joh, Jin Hee Bae, Sun Ja Kim, Tae Hun Chung, Jin Woong Chung

**Affiliations:** 1 Department of Biological Sciences, Dong-A University, Busan, Korea; 2 Department of Physics, Dong-A University, Busan, Korea; Kwangwoon University, REPUBLIC OF KOREA

## Abstract

Plasma activated medium (PAM) was prepared by a bipolar microsecond-pulsed atmospheric pressure plasma jet (APPJ) array source and was utilized for cancer cell treatment. APPJ array-produced plasma were characterized. APPJ array treatment of three different solutions (deionized water (DW), HBSS (serum-free Hanks’ balanced salt solution), and DMEM (Dulbecco’s Modified Eagle Medium) + 10% FBS (fetal bovine serum)) were performed to induce the changes in the concentration of reactive oxygen and nitrogen species (RONS) as functions of the operating parameters. Human cervical cancer cells (HeLa) injected with plasma-treated media were investigated for changes in cell viability using MTT assay. It was observed that PAM-induced ROS can regulate the protein expression associated with mitochondria, and PAM causes apoptosis through Cyto C/JNK/p38 signaling on human cervical cancer cells.

## Introduction

Non-thermal atmospheric pressure plasmas (or cold atmospheric plasmas (CAPs)) have received considerable attention because of their widespread applications for inactivation of microorganisms, sterilization, and cancer therapy [1–3]. There are numerous studies showing that CAPs are effective in treating cancer cells both in-vitro and in-vivo. Living cells and tissues were directly exposed to plasma treatment, or the cell culture medium or buffer solution was first exposed to plasma treatment (so-called plasma-activated medium (PAM)) and then added to medium-free cells [4–8]. Indirect treatments with PAM demonstrate similar bactericidal and or cytotoxic effects as that of direct plasma exposure, but facilitate flexibility and precision of delivery with potentially gentler conditions as may be demanded with *in-vivo* conditions [7, 8].

Since most plasma-generated species are highly reactive and short-lived, PAM mainly contains long-lived species from CAP, such as H_2_O_2_, NO_2_^-^, NO_3_^-^, and O_3_ [4–6, 9]. There is very strong evidence that RONS contained in PAM are responsible for apoptosis induction in tumor cells. It is of interest and challenging that a PAM with a RONS composition of such an apparently low complexity can induce significant antitumor effects in many tumor cells in vitro and in vivo [10]. H_2_O_2_, NO_3_^-^, and NO_2_^-^ present in PAM play a key role in the induction of cell death in cancer cells. These RONS can further favor the formation of peroxynitrite (ONOO^-^), which has a high potential to induce lipid peroxidation [11]. Nitrate/nitrite anions can be the target of short lifetime ROS such as OH leading to the formation of ONOO^-^ [12]. It is mostly ONOO^-^, NO_2_, N_2_O_3_ that will induce protein and DNA damage, and they might be also responsible for cytotoxic effect of PAM [13].

It was suggested that a synergistic effect between PAM-contained H_2_O_2_, and NO_2_^-^ is responsible for selective antitumor action of PAM [4, 9]. The interaction of these compounds was shown to result in the formation of primary singlet oxygen (_1_O^2^) that caused local inactivation of membrane-associated catalase on tumor cells [10]. However, the cell death pathways at a molecular level have not yet been elucidated. Furthermore, it was found that there is an optimum dose of PAM to induce significant cancer cell apoptosis while keeping minimum damage to normal cells [14].

The quality and activity of the PAM can be affected by various parameters, such as discharge type and power, composition of gas mixture and gas flow rate, and the type of treated medium [15]. Some recent reports have proposed various types of APPJ array to enhance the treatment area, thus providing a larger and more homogeneous surface treatment [16–19]. The use of plasma jet array enables us to achieve a large area plasma treatment with enhanced discharge power in preparing PAM. In our previous work [19], we explored the possibility of APPJ array driven by a bipolar microsecond-pulsed high voltage to generate PAM for cancer therapy. The effect of different parameters (applied voltage, gas flow rate, and pulse repetition frequency) on the plasma properties and the production of RONS in the PAM was studied to obtain optimal plasma condition for preparing PAM, and finally, the APPJ array-generated PAM was applied to cancer cells in-vitro to assess its applicability for cancer therapy.

Furthermore, in preparing PAM, the volume of media is also important parameter for the production of RONS [20]. If a larger volume of PAM containing a comparable concentration of RONS could be produced at a fixed power level, it would be more efficient way for preparing PAM. This paper explores the dependence of RONS production on the volume of media. In addition, since a more detailed mechanism behind the cell death by PAM needs to be clarified, this paper will discuss that issue, providing a possible pathway to apoptosis via PAM.

## Materials and methods

### Plasma source and characterization

**Fig 1:**
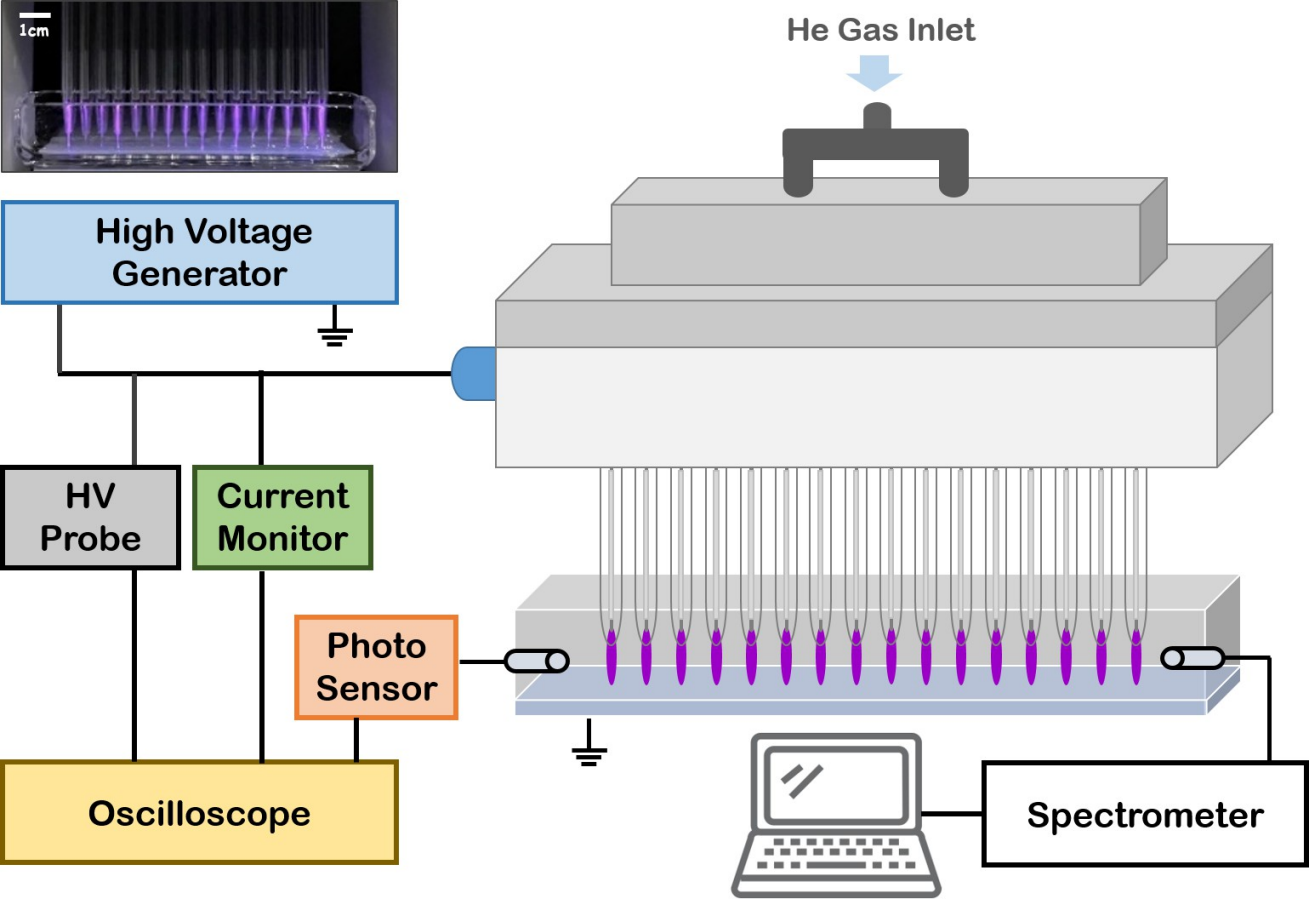
(a) Photograph of the plasma plumes from the jet array contacting a medium in a liquid container, and (b) the schematic of the experimental setup.

Fig 1 depicts a schematic of the jet array source driven by a microsecond-pulsed bipolar high voltage and the plasma plume touches a medium in a liquid container. The details of the jet array are found in our previous papers [18, 19]. The helium was used as a working gas. The voltage and the current were measured using high voltage probe (PPE 20kV LeCroy) and current probe (3972 Pearson). The optical emission spectra were obtained using a fiber optic spectrometer (USB-2000+XR1-ES Ocean Optics). The ozone generation in the gas phase was detected using an ozone detector (2B Technologies Model 202) that is based on the absorption of UV light at 254 nm.

### Quantization of hydrogen peroxide (H_2_O_2_), nitrate (NO_3_^-^), nitrite (NO_2_^-^), and ozone (O_3_) in the PAM

OH radical can be formed by the reaction of an oxygen atom with an H_2_O molecule at the liquid surface or from the solvation of gaseous OH produced in the gas phase of plasma giving rise to the production of aqueous OH radicals [14]. OH radicals, acting as a precursor of H_2_O_2_ in PAM, is an important ROS with a strong oxidizing ability in an aqueous environment and at the interface between gas and liquid phases [20]. The concentrations of nitrate/nitrite anions in PAM seem to be affected by the composition of the different media. Both nitrogen species are formed in plasma treated media through the dissolution of nitrogen oxides formed in gas phase [14]. In this work, we consider three different solutions; deionized water (DW), HBSS (serum-free Hanks’ balanced salt solution, and DMEM (Dulbecco’s Modification of Eagle’s Medium) + 10% FBS (Fetal bovine Serum).

Nitrite concentration was determined using the Griess reagent (Molecular Probes). In liquids, nitric oxide is converted into nitrate/nitrite anions [21]. The Griess reaction can provide a good measure of the concentrations of these nitrogen oxides [21]. Experimental monitoring of RONS in liquid is crucial for model benchmarking and provides the most direct information on the reactive species present in the liquid [22]. UV-vis spectrophotometry is an appropriate method for detection of RONS in aqueous solutions [23]. The concentrations of the long-lived reactive species such as hydrogen peroxide (H_2_O_2_), nitrate (NO_3_^-^), ozone (O_3_) in the PAM were determined spectrophotometrically on the PhotoLab 7600 (WTW, Germany) according to instructions provided by the manufacturer [16].

### In-vitro cell experiment

#### Cell culture

HeLa, A549 and T24 cells were grown in Dulbecco Modified Eagle Medium (Capricorn Scientific, Ebsdorfergrund, Germany) and H1299 were incubated in Roswell Park Memorial Institute with L-glutamine media (RPMI, Capricorn Scientific, Ebsdorfergrund, Germany). The media were supplemented with 10% fetal bovine serum and 1% penicillin/streptomycin (Capricorn, Scientific, Ebsdorfergrund, Germany). The cells were incubated in a humidified atmosphere with 5% CO_2_ at 37°C.

#### Cell viability assay

Cell viability was measured using the MTT (Duchefa, Haarlem, Netherlands) assay. HeLa cells were seeded in 96 well plate at 1 × 10^4^ cells/wells. Plasma activated medium were removed after incubation for 24 hours and replaced with 100 μL MTT solution (5 mg/mL). At the end of treatment, MTT solution was removed and added the 100 μL DMSO for detect absorbance at 550 nm. The cell viability (%) was calculated (O.D. of treated cells / O.D. of non-treated cells × 100).

#### Cell apoptosis assay

MUSE Annexin V & Dead Cell kit (Luminex, Texas, USA) was used to detect apoptosis. HeLa cells were treated plasma activated medium for 24 hours and were collected in microtube. After removing the supernatant, 100 μL Annexin V & Dead Cell reagent was added. The mixture was incubated for 20 minutes at room temperature in dark station. Finally, cell apoptosis rate (%) was measured using Muse Cell Analyzer and software.

#### Mitochondrial superoxide measurement

Mitochondrial superoxide was confirmed using MitoSOX (Invitrogen, Carlsbad, USA). HeLa cells were cultured on a gelatin-coated coverslip. After 2 hours of plasma activated-medium treatment, 5 μM MitoSOX was treated for 10 minutes. Then, the HeLa cells were treated with 10 μL Hoechst 33342 (10 μg/1 mL) was treated for 30 min and confirmed by confocal microscopy.

#### Mitochondrial potential measurement

We used the Muse MitoPotential Kit (Luminex, Texas, USA) for measure the proportion of depolarized mitochondria following the manufacturer’s protocol. First, HeLa cells were incubated with plasma activated medium for 6 hours. Next, the cells were harvested and added 95 μL of Muse MitoPotential working solution to 100 μL of cells. After incubation at 37°C for 20 minutes, added of 5 μL Muse 7-AAD reagent into each microtube. Then, we mixed thoroughly microtube and run on Muse Cell Analyzer.

#### Western blot

HeLa cells were treated with plasma-activated medium for 2 hours. Next, cells were collected and lysed using RIPA buffer (iNtRON, Seongnam, Korea). Each protein was separated by size through SDS-PAGE and transferred to PVDF membrane (Millipore, Billerica, MA, USA). The membranes were incubated with specific primary and secondary antibodies. We measured the level of protein expression using ECL reagent (Dongin, Seoul, Korea) and X-ray film (Fuji Film, Tokyo, Japan).

## Results and discussion

### Characteristics of electrical discharges

**Fig 2:**
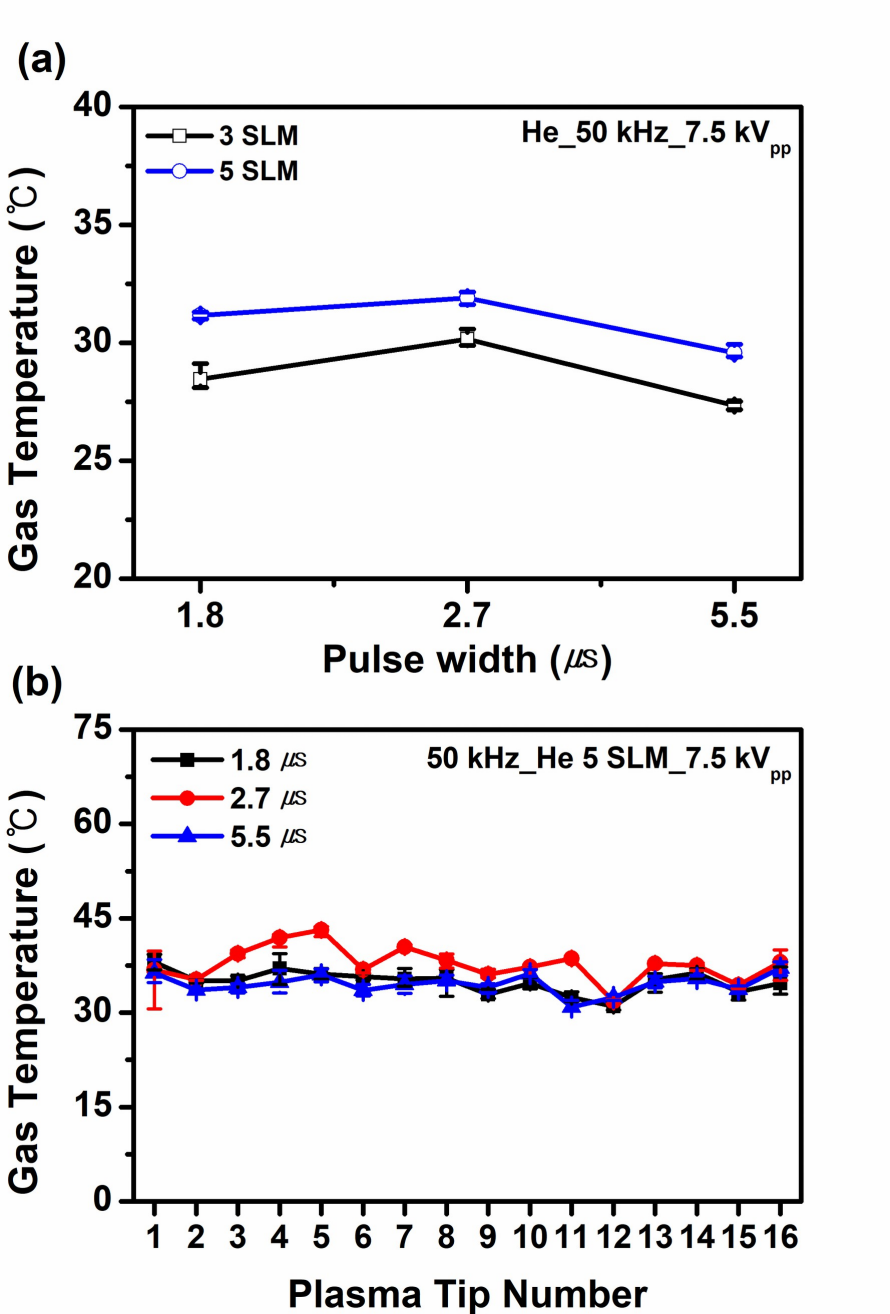
(a) Gas temperature as a function of pulse width at two different gas flow rates (3 SLM and 5 SLM). (b) Measured gas temperature at each nozzle of the jet array as a function of the pulse width of applied voltage.

The plume temperature that was measured using a fiber optic temperature sensor (Luxtron, M601-DM&STF) remained less than 42.5°C at the applied voltage of 7.5 kV_pp_. As can be seen in Fig 2, with an increase in the gas flow rate, the gas temperature decreases slightly. Fig 2(B) shows plume temperatures measured at each nozzle of the jet array for three different pulse widths (1.8, 2.7, and 5.5 μs) of applied voltages. It is found that a slightly higher gas temperature is observed at the pulse width of 2.7 μs and it exhibits a spatial uniformity within a tolerable limit.

**Fig 3:**
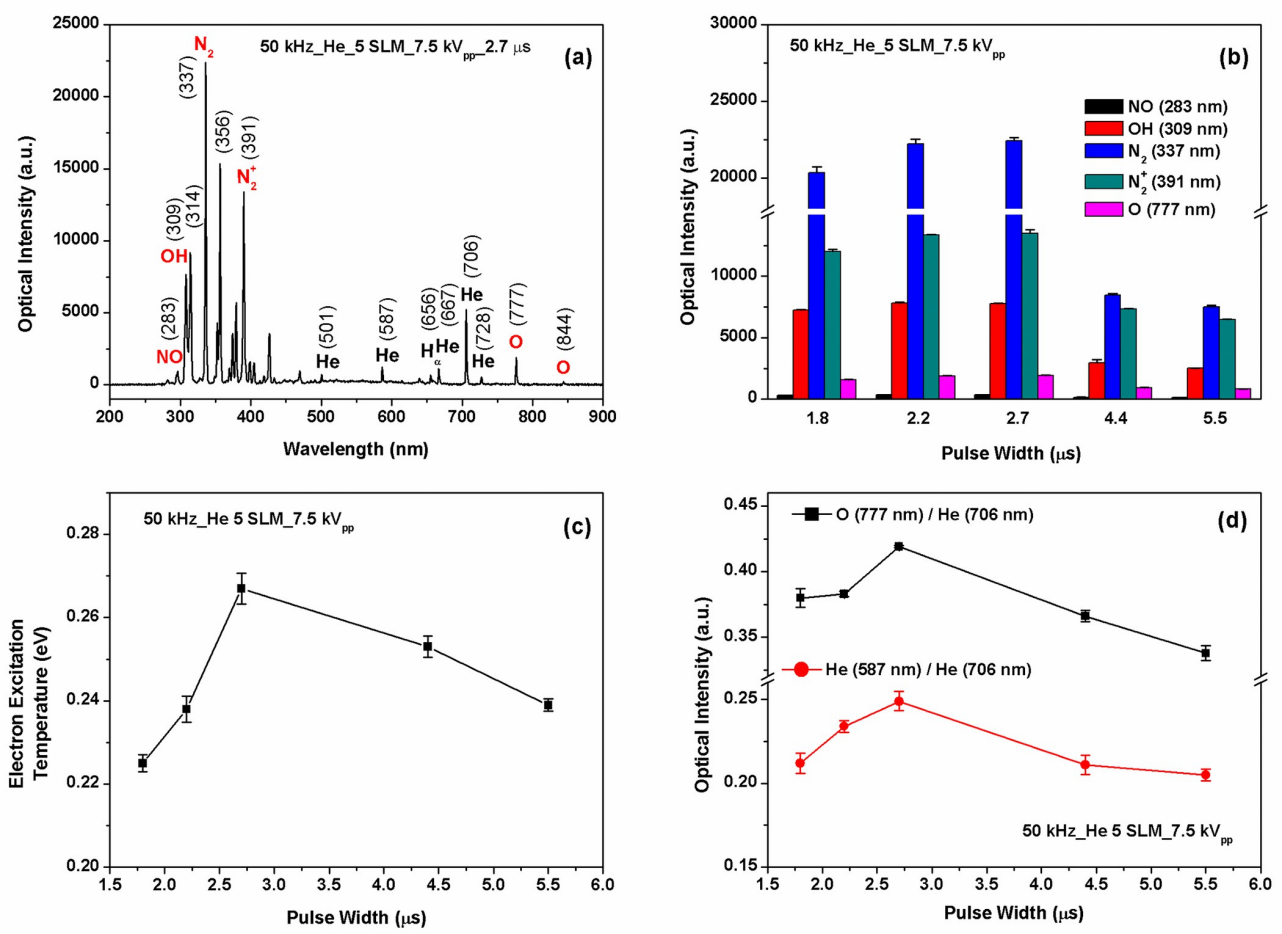
(a) Typical optical emission spectrum from the jet array plasma. (b) The peak intensities of important lines (N_2_^+^, N_2_, OH, O, NO) as a function of the pulse width. (c) Excitation temperature was estimated based on Boltzmann plot at various pulse widths (1.8 ─ 5.5 μs). (d) The intensity ratios of two atomic lines He (587 nm)/He (706 nm) and O (777 nm)/He (706 nm) at several different pulse widths (1.8 ─ 5.5 μs).

Fig 3(A) illustrates the optical emission spectrum for the jet array. The strongest emission peaks are associated with molecular nitrogen and ionic molecular nitrogen. The N_2_^+^ first negative system at 391 nm (B^2^∑_u_^+^ → X^2^∑_g_^+^) is attributed to Penning ionization and charge transfer followed by direct electron-impact excitation [24]. Along with this, the N_2_* second positive system (C^3^ Π _u_ → B^3^ Π _g_), clearly observed at 315, 337, 357, 375, and 380 nm, indicates the fact that main part of the absorbed energy from the electric field is transferred from metastable helium to molecular nitrogen for their excitation and ionization [24]. The hydroxyl (OH) radical at 309 nm is produced via dissociative electron attachment to the water vapor molecules. The emission line at 656 nm corresponds to the H_α_ line. The He 706 nm emission [3s ^3^S → 2p ^3^P] indicates the presence of energetic electrons. The 777 nm and 844 nm lines originate from reactive excited O atoms such as O [3p ^5^P] and O [3s ^5^S] [25]. Metastable helium can dissociate the oxygen molecules and excite atomic oxygen to the excited state. It should be noted that the APPJ array produce significantly enhanced intensity levels of OH (309 nm) band and N_2_^+^ band (391 nm and 427 nm), and relatively high level of intensities from reactive radicals such as NO, O, and H_α_. Especially, since OH radical induces an important biological response [4–8, 26, 27], high levels of OH generated in our APPJ array may provide a significant advantage in biomedical applications including cancer therapy.

Fig 3(B) shows the peak intensities of important lines (N_2_^+^, N_2_, OH, O, NO) at various pulse widths (1.8 ─ 5.5 μs). These lines or bands imply the existence of N_2_*, OH, O, NO radical in the plasma-liquid interface and these radicals are important for modification of liquid characteristics [24]. The figure indicates that the peak intensities of N_2_^+^, N_2_, OH, O, NO lines or bands were the highest at the pulse width of 2.7 μs. (The single APPJ driven by the same power supply exhibited a similar phenomenon [23]).

The characteristic spectral lines of helium were chosen to determine the excitation temperature (*T*_exc_) under a Boltzmann approximation [28, 29]. Fig 3(C) presents the excitation temperature of the jet array as a function of the pulse width. The excitation temperature becomes maximal at the pulse width of 2.7 μs. In our previous work [19], we observed that with an increase in gas flow rate, the *T*_exc_ rises and reaches a maximum at 8 SLM, and then declines slightly.

The intensity ratio of two atomic lines of He, 587 nm (3d ^3^D → 2p ^3^P) and 706 nm (3s ^3^S → 2p ^3^P), is closely related to the ratio of upper state’s density, which depends on the electric field or the electron energy [30]. Assuming that the ratio of excitation coefficient of He to 3s ^3^S and that of O to 3p ^5^P remains constant with the other control parameters unchanged, we can estimate the effect of the pulse width on O density by taking the emission intensity ratio of O (777 nm) to He (706 nm). Fig 3(D) illustrates the intensity ratios of He (587 nm) to He (706 nm) and the intensity ratios O (777 nm) to He (706 nm) as a function of the pulse width. It is observed that these ratios exhibit a similar trend of change to those of the excitation temperature. A possible explanation for this trend can be that oxygen atom density is proportional to the electron energy, being enhanced at the pulse width of 2.7 μs.

When pulse width is too large, the voltage-off duration becomes short, the decay of charged species and excited neutrals becomes slow, and the accumulated positive space charges remains longer on the tube, resulting in the increase in the reverse electric field, thus the decrease in the effective electric field. On the other hand, when the pulse width is too small, the ionization front is prematurely terminated by the falling edge of the applied voltage pulse, and thus, the energy dissipation becomes small, resulting in weak discharges. Therefore, the pulse width influences the effective electric field, and thereby the discharge current (and also the discharge power), and finally determines the production of reactive species in gas phase [20, 25, 30]. This is also agreeable with the results that a larger excitation temperature (thus higher electron energy and optical emission intensities) is observed at the pulse width of 2.7 μs (Fig 3(B) and 3(D)).

### Ozone concentration in the gas—and liquid phase

**Fig 4:**
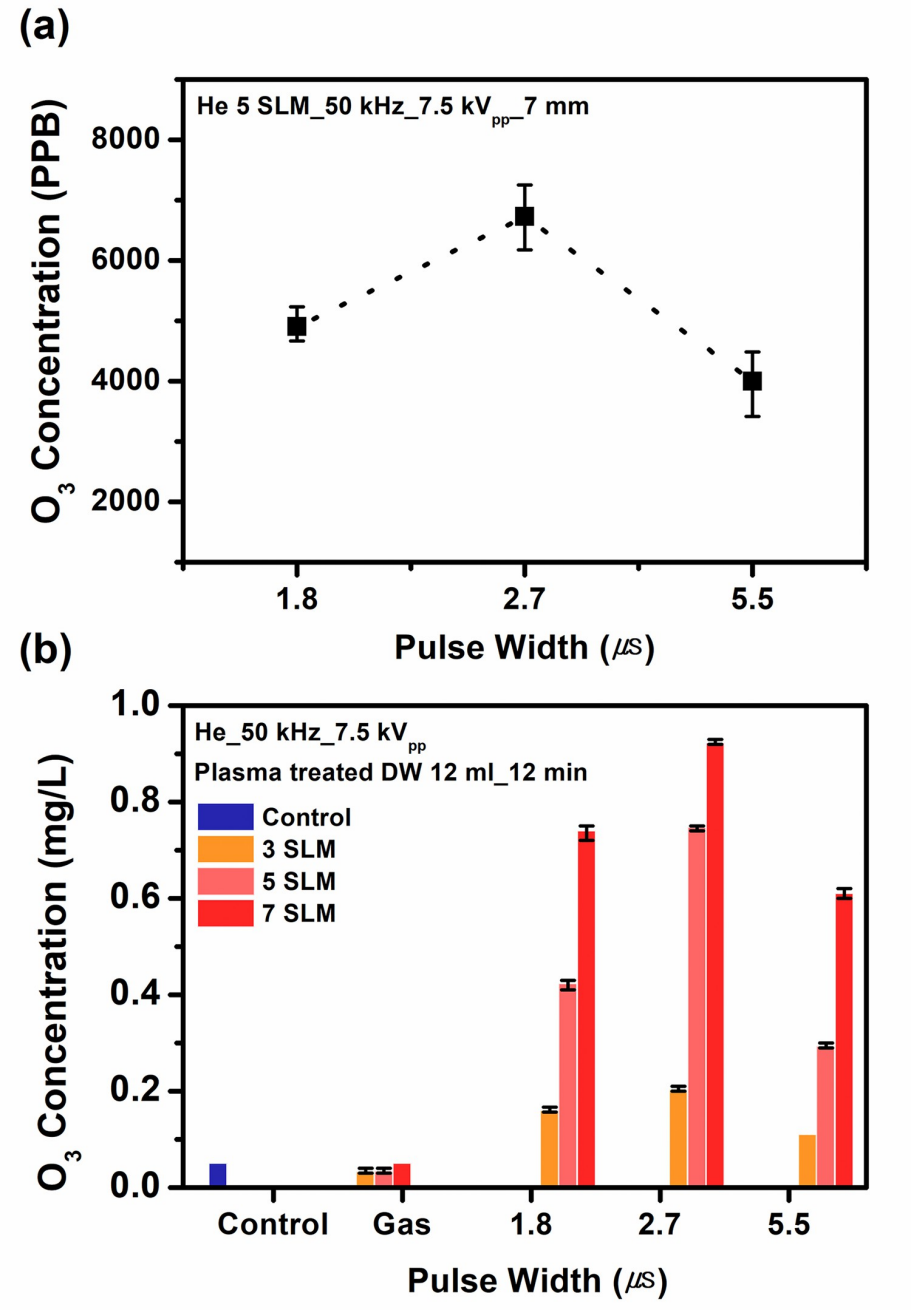
Ozone concentration as a function of pulse width: (a) in the gas phase, (b) in the PAM. Three different gas flow rates (3 SLM, 5 SLM, and 7 SLM) are considered.

APPJ array produces a significant amount of ozone (O_3_), which is known to have strongly harmful effect on cells. Ozone play some role in several ways in the formation of RONS in aqueous media and has received much attention as a strong oxidant with long lifetime in biomedical applications [23]. O_3_ is the precursor for much stronger oxidants such as the hydroxyl radical (•OH) [31]. Ozone is mainly produced via the recombination reaction of an oxygen atom and an oxygen molecule in the gas phase, and subsequently transported to the liquid phase by solvation. Fig 4(A) presents the O_3_ concentrations in the gas phase at the pulse widths of 1.8, 2.7, and 5.5 μs for varying gas flow rate. The O_3_ density tends to continuously increase with the gas flow rate, and has the highest value at the pulse width of 2.7 μs, where the atomic oxygen density is quite high. It should be noted that the jet array seems to have very high O_3_ concentrations in the gas phase, far beyond the safety limit, which may restrict its use to the indirect treatment. In our previous study [23], we observed that the ozone generation became slightly lower as the pulse width was increased from 2.7 to 5.5 μs, and that in microsecond-pulsed APPJs, a lower pulse width might be advantageous for production of the singlet oxygen molecule and negative molecular oxygen ions which could be utilized for ozone formation. Fig 4(B) presents the ozone concentrations in the PAM. The dependence of [O_3_] on the pulse width remains to exhibit the same trend, but the ozone concentration in the treated DW is much lower than [H_2_O_2_] in the treated DW (see Fig 6). That may be because that H_2_O_2_ has much higher dissolution rate than O_3_ [32].

### Generation of hydrogen peroxide, nitrite, and nitrate

**Fig 5:**
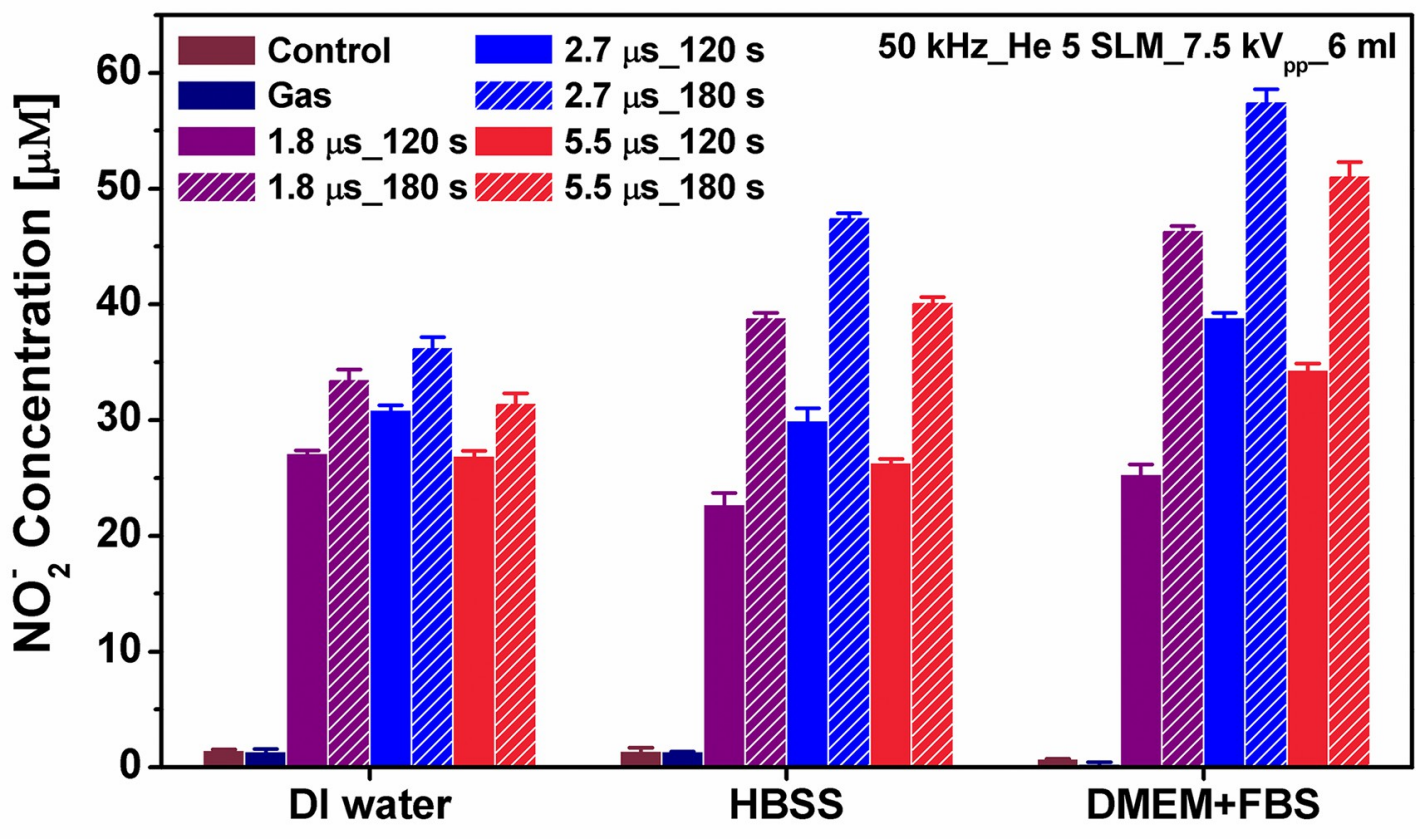
Nitrite concentrations in different media (DW, HBSS, and DMEM) immediately after the plasma exposure as a function of the pulse width of the applied voltage and plasma exposure time. Here, the applied voltage *V*_*a*_ = 7.5 kV_pp_, the gas flow rate 5 SLM, the plasma exposure time was 120 and 180 sec.

APPJ array treatment of three different media (deionized water (DW), HBSS (serum-free Hanks’ balanced salt solution), and DMEM+FBS) were performed to induce the changes in the concentration of RONS as functions of the operating parameters. Fig 5 shows the results of the nitrite (NO_2_^-^) quantitation assay performed on three different media after plasma treatment. For the quantification of the nitrite concentrations, a standard procedure of Griess assay was performed [19]. In our previous work, we observed that the nitrite concentration increased after plasma exposure with increasing applied voltage and increasing gas flow rate [19]. As shown in Fig 5, no significant difference is observed in gas-treated media (control). Nitrite concentration was found to increase with plasma exposure time and to be higher at the pulse width of 2.7 μs. Nitrite concentration in DMEM was higher than those in both HBSS and DW. DMEM contains a higher concentration of amino acids and vitamins, as well as additional supplementary components, while HBSS is simply composed of various salts. Plasma might react with amino acid to form NO_2_^-^ components, which may provide a possible explanation for higher NO_2_^-^ concentration in DMEM compared to those in DW and HBSS.

**Fig 6:**
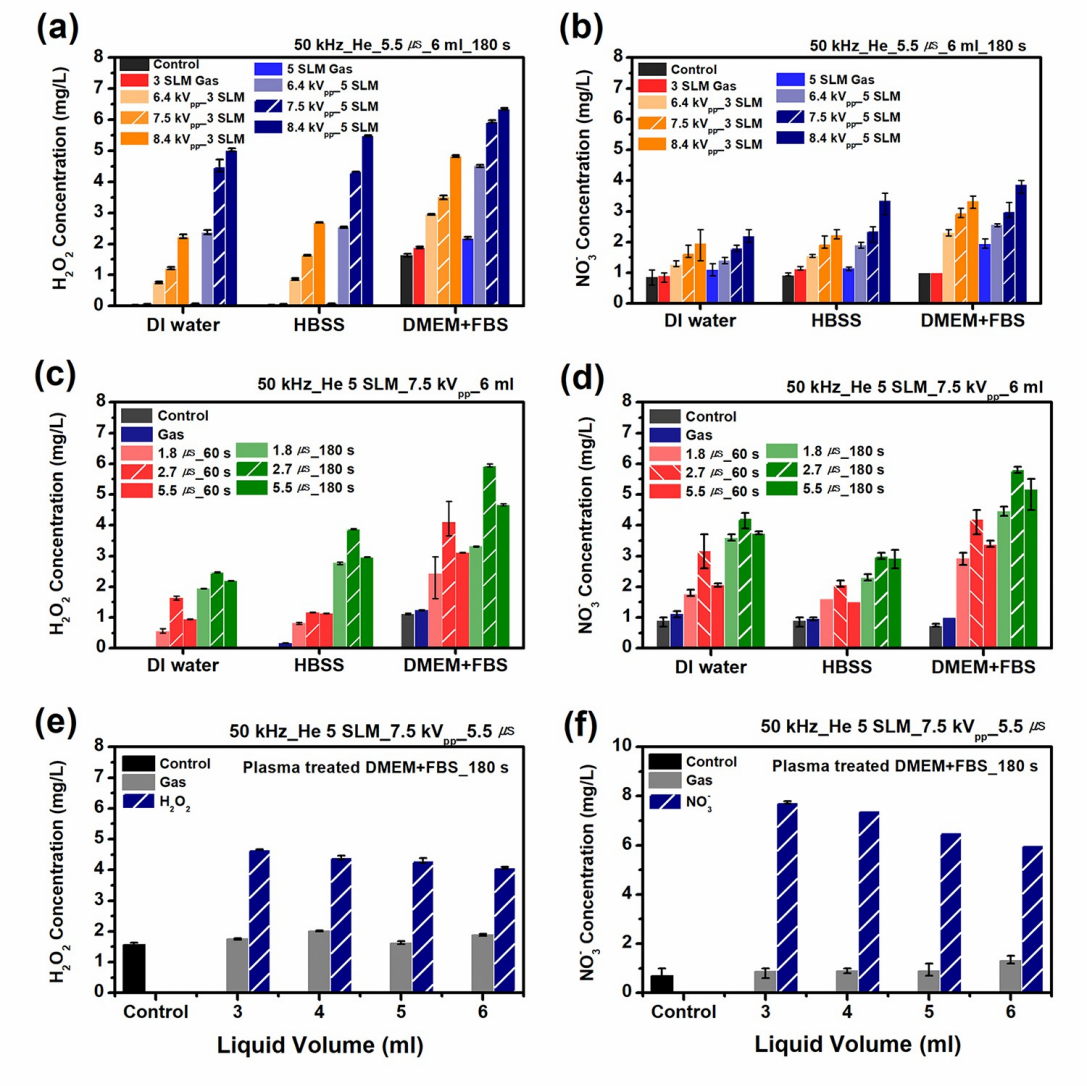
Concentrations of H_2_O_2_ and NO_3_^-^ produced in the plasma-treated media (DW, HBSS, and DMEM) at different conditions: (a) and (b) present the measured concentration of H_2_O_2_ and NO_3_^-^ as functions of the applied voltage and the gas flow rate, respectively. (c) and (d) present the measured concentration of H_2_O_2_ and NO_3_^-^ as a function of the treatment time at three different pulse widths (1.8, 2.7, and 5.5 μs), respectively. (e) and (f) present the dependence of [H_2_O_2_] and [NO_3_^-^] on the volume of media (DMEM + 10% FBS), respectively. In (e) and (f), [H_2_O_2_] and [NO_3_^-^] were measured as a function of media volume in the range from 3 ml to 6 ml. The grey bar represents the gas-only treated case.

In order to quantify the stable RONS in PAM, the concentrations of H_2_O_2_ and NO_3_^-^ produced in the plasma-treated media (DW, HBSS, and DMEM) at different conditions are measured using UV-vis spectrophotometry. In Fig 6(A), [H_2_O_2_] and [NO_3_^-^] are determined after plasma treatment (3 min) with 6 ml of media. As shown in the figure, [H_2_O_2_] and [NO_3_^-^] increase with the applied voltage and the gas flow rate. The formation of H_2_O_2_ is favored by the presence of the OH radical, the main route of H_2_O_2_ generation in the liquid being by the recombination of OH radicals in the gas phase and subsequent diffusion in the liquid phase [24]. With increasing gas flow rate, the gas temperature decrease (while the electron temperature slightly increases; in the case of jet array, the gas flow is distributed into multiple jets and until up to some threshold flow rate, the electron temperature increases with gas flow rate), thereby the production of H_2_O_2_ (OH + OH → H_2_O_2_) is enhanced, while the destruction of H_2_O_2_ (e + H_2_O_2_ → OH + OH^-^) is slightly inhibited, furthermore, the reaction OH + H_2_O_2_ → H_2_O + HO_2_ is oppressed [33]. The net result is that we would have a higher amount of [H_2_O_2_] with increasing gas flow rate. Also, for a similar reason mentioned in the discussion on the generation of NO_2_^-^ in Fig 5, it was observed that [H_2_O_2_] in DMEM was higher than those in HBSS and DW. Fig 6(A) and 6(B) show that the [NO_3_^-^] has little lower values than those of [H_2_O_2_], but this ordering is in contrary to the case of PBS (phosphate-buffered saline) media [6, 34, 35]. As expected, [NO_3_^-^] increases with both the applied voltage and the gas flow rate. However, [NO_3_^-^] has quite a different dependence on the gas flow rate. With increasing gas flow rate, as before, the electron temperature increases, therefore the formation of metastable state N_2_*(A) is enhanced. NO is formed by various reactions (N + OH → NO + H; N + O → NO; N + O_2_ → NO + O), NO_2_ is formed by the reactions (O_3_ + NO → NO_2_ + O_2_; 2NO + O_2_ → 2 NO_2_) [36]. Although very complicate pathways are involved, we note that with increasing gas flow rate, the production of N_2_*(A) is increased and the formation of NO_x_ is enhanced [36], which might result in the larger amount of HNO_x_ (the precursor of NO_3_^-^ and NO_2_^-^) in the gas phase. This effect surpasses the gas temperature effect (which might act negatively) acting on various chemical reactions mentioned above. This can explain the reduction of the proportionality of [NO_3_^-^] change with respect to the gas flow rate compared with those of [H_2_O_2_].

Fig 6(C) and 6(D) present [H_2_O_2_] and [NO_3_^-^] as a function of the treatment time at three different pulse widths (1.8, 2.7, and 5.5 μs), respectively. We observe that the pulse width of 2.7 μs produces the highest production rate of [H_2_O_2_], and the RONS concentration increases linearly with the treatment time. This may be explained by the fact that a lower pulse width is advantageous for production of short-lived radicals, which could be utilized for the formation of the long-lived reactive species [23]. This observation demonstrates that complicated chemical reactions occurrs at the gas–liquid interface in generating reactive species, and the secondary products may be also dependent on the pulse width of the applied voltage [6, 23]. Also, this agrees with the effects of the pulse width on the quantity of OH obtained by the UV absorbance and the TA method [23], confirming that H_2_O_2_ is mainly formed via OH recombination. Fig 6(D) shows [NO_3_^-^] as a function of the treatment time at different pulse widths. This can also be explained in a similar way to the [H_2_O_2_] case.

Fig 6(E) and 6(F) present the dependence of [H_2_O_2_] and [NO_3_^-^] on the volume of media (DMEM + 10% FBS), respectively. The [H_2_O_2_] and [NO_3_^-^] were measured as a function of media volume in the range from 3 ml to 6 ml. It can be seen that smaller volumes can be associated with a little higher [H_2_O_2_] and [NO_3_^-^]. These dependences suggest that the species responsible for the changes in [H_2_O_2_] and [NO_3_^-^] are only formed at the plasma-liquid interface and then transferred to the liquid, and that the production rate of the species does not depend on the treated volume [24]. These results strengthen the assumption that at least for our set-up, the treated volume does not influence the production rate of the species. Because multiple plasma plumes touch the treated media in the APPJ array considered in this study, RONS concentrations exhibit a slighter decrease with increasing volume of media compared with the case (not shown in the figures) of microwave-excited jet having a single plume impinging on the liquid [37].

### Cell viability of the human cervical cancer cells

**Fig 7:**
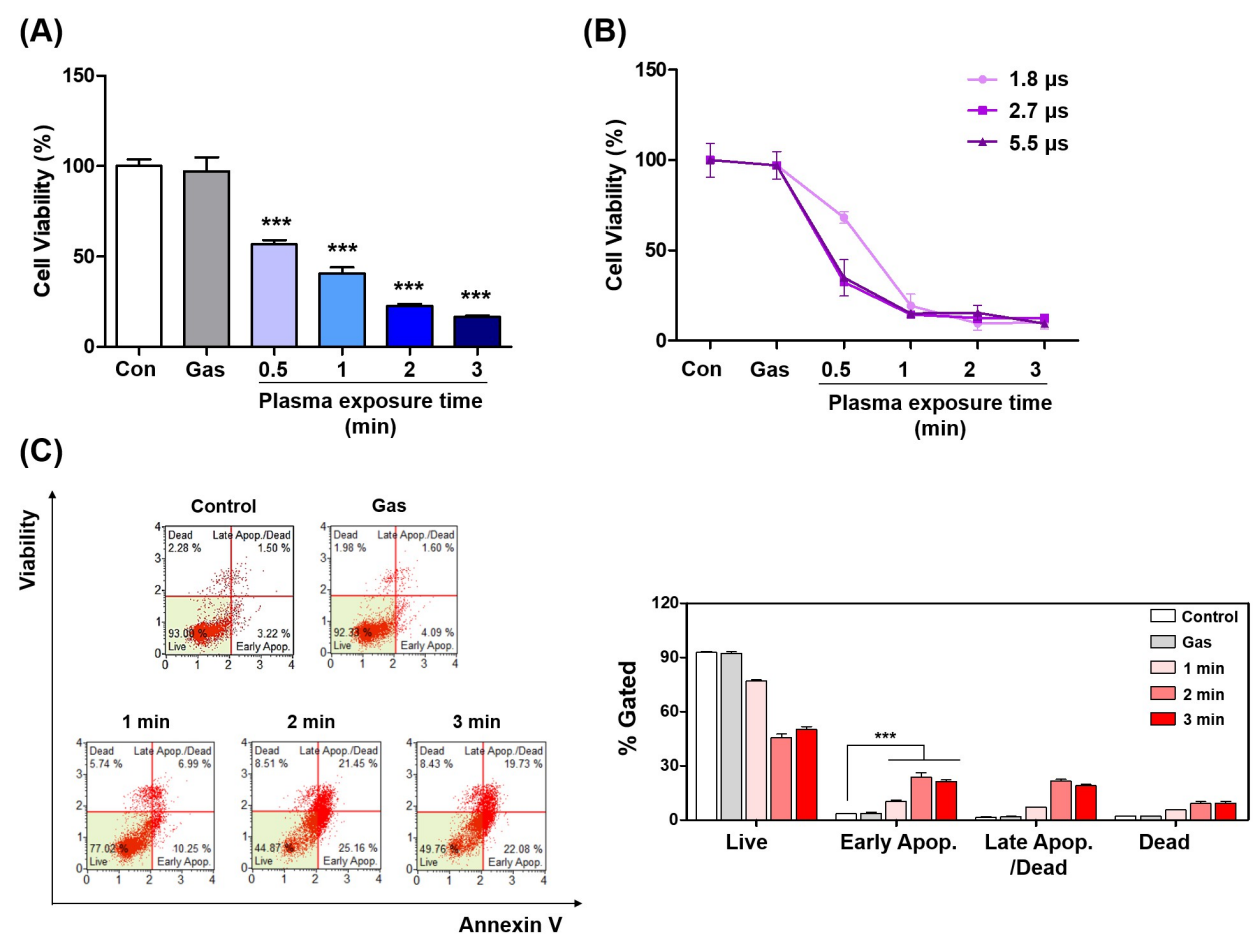
Plasma activated medium (PAM) induces apoptosis in human cervical cancer cells. Cell viability measurement in HeLa cell lines at 24 h after PAM treatment. (A) Plasma conditions; the gas flow rate 5 SLM. Medium was exposed by plasma operated with the applied voltages at the pulse width of 2.7 μs for 0.5, 1, 2, and 3 minute. (B) Plasma conditions; the gas flow rate 5 SLM, applied voltage 7.5 kV_pp_, plasma exposure time 0.5, 1, 2, and 3 minute. (C) PAM induces apoptosis in HeLa cells. The percentage of the apoptotic cells was measured using the MUSE Cell Analyzer. Medium irradiated with plasma operated with the applied voltages at the pulse width of 2.7 μs was applied to HeLa cells for 24 hours. Bar graph indicated the quantitative analyses of each population. Data represent the mean ± S.D. of n = 3 samples; ***P<0.001.

To test the hypothesis whether PAM could induce the cell death in HeLa, we checked the cell viability using MTT assay. The result indicated that PAM reduced the cell viability in all conditions (Fig 7A). Although the pulse width of applied voltage had little effect on cell viability, the plasma exposure time affected the decline of cell viability (Fig 7B). To check whether PAM can induce cell death in other types of cancer cells, human lung cancer cells (H1299 and A549) and human bladder cancer cells (T-24) were treated with PAM prepared at different pulse width (of applied voltage) and plasma exposure times. PAM reduced the viability of these cell lines with a similar dependence on the pulse width and plasma exposure times to that of HeLa cell. However, the influence of pulse width on the viability of A549 cells was not significant. (see the S1 File) In our previous study [37], we observed that PAM induced lower cell cytotoxicity in normal cells in contrary to higher cell cytotoxicity (and higher intracellular ROS) in cancer cells when PAM under the same conditions was treated with normal cells and cancer cells. Other studies have reported that normal cells are less responsive to exogenous ROS by less aquaporins and more catalases [38].

### Apoptosis by PAM causes by mitochondrial superoxide

**Fig 8:**
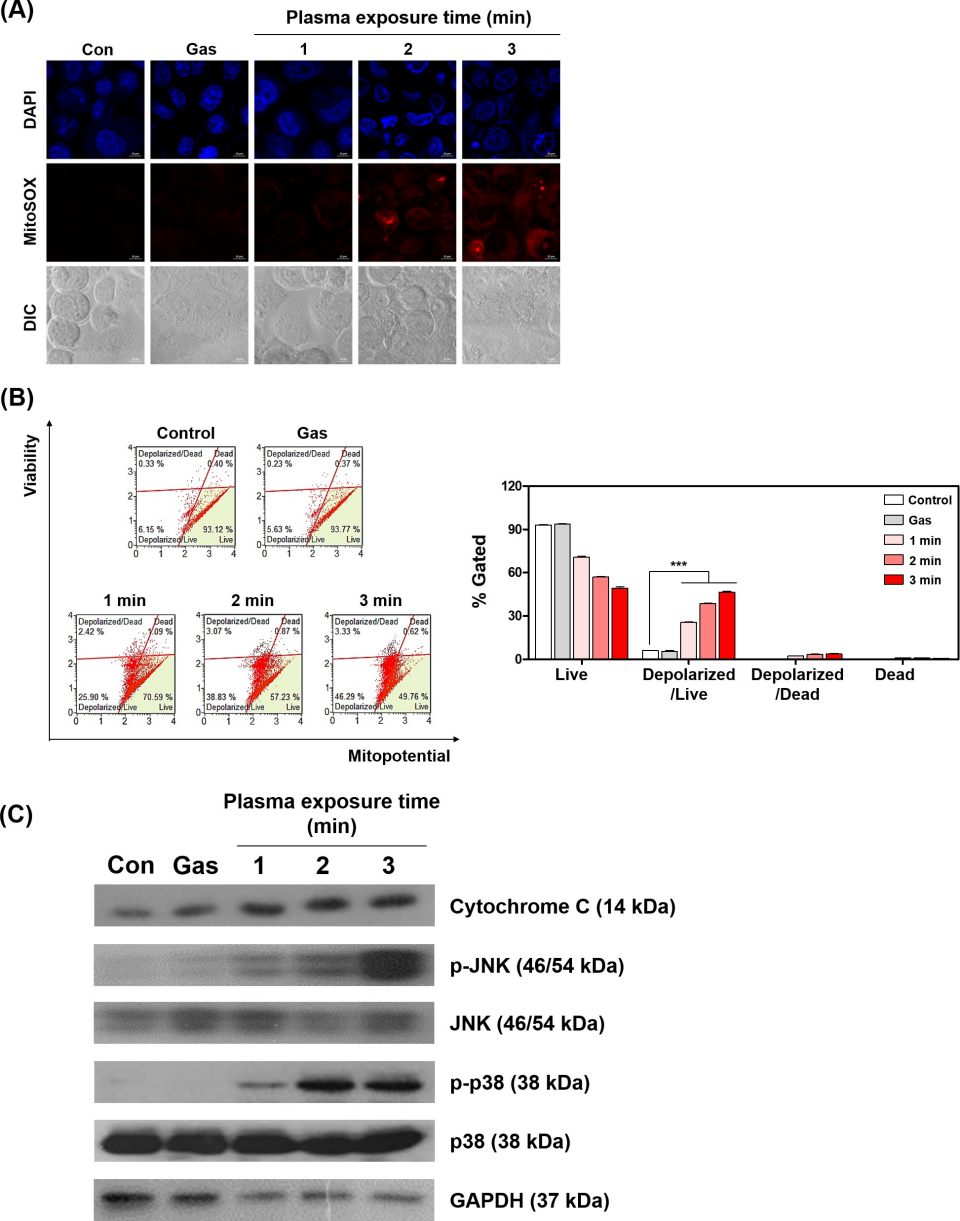
Plasma activated medium (PAM) induces mitochondrial depolarization through upregulating mitochondria ROS. (A) Levels of mitochondrial superoxide was observed by DAPI and MitoSOX fluorescence using confocal microscopy. Representative fluorescence images show the staining of nuclei counterstained with DAPI (blue) and MitoSOX (red). HeLa cells were treated with PAM (pulse width 2.7 μs, incubation time 2 h). (B) Mitochondria potential was determined using the MUSE Cell Analyzer. Growth medium exposed to plasma was incubated with HeLa cells for 6 hours. Bar graph indicated the quantitative analyses of each population. (C) Western blotting analysis of Cytochrome C, phospho-JNK, JNK, phospho-p38 and p38 expression in HeLa cells treated with PAM (pulse width 2.7 μs, incubation time 2 h). Data represent the mean ± S.D. of n = 3 samples; ***P<0.001.

In our previous research [37], we reported that PAM increase apoptosis in human lung cancer cells. A similar trend was observed in HeLa cells. When the HeLa cells were treated with PAM and incubated for 24 hours, total apoptotic rate was increased (Fig 7C). Apoptosis has been known to cause by various cellular response [39]. Among them, apoptosis caused by ROS is highly influenced by mitochondria [40, 41]. Adachi et al. reported a mitochondrial dysfunction-mediated caspase-independent signal pathway in the cases of cancer cell injuries by PAM [41]. We investigated whether PAM-induced apoptosis was associated with mitochondria. Mitochondria are known to increase superoxide levels in an oxidative environment. We measured mitochondrial superoxide using MitoSOX. After PAM treatment, the level of mitochondrial superoxide was observed to increase depending on the plasma exposure time (Fig 8A). This result strongly supports that increased superoxide leads to dedifferentiation of mitochondria. The depolarization also increased depending on plasma exposure time (Fig 8B). Mitochondrial apoptosis is associated with the release of cytochrome C [42]. These results show that the expression of cytochrome C was increased depending on plasma exposure time. In our previous study, we confirmed that PAM induces phosphorylation of JNK in human lung cancer cells [37]. Also, JNK activation has been reported to cause mitochondrial dysfunction [43]. In the present study, PAM induced JNK phosphorylation. In addition, this increase also induced the phosphorylation expression of p38 (Fig 8C). JNK and p38 are common sub-signals regulated by MAPK. As a result, PAM-induced ROS can regulate the protein expression associated with mitochondria. Taken together, PAM causes apoptosis through Cyto C/JNK/p38 signaling on human cervical cancer cells.

## Conclusion

An APPJ array driven by microsecond-pulsed bipolar high voltages was characterized with varying operating parameters including the pulse width of the applied voltage. Around the pulse width of 2.7 μs, the discharge current, power, and the light emission intensity were observed to be high. The APPJ array can achieve a higher discharge power than a single jet at the same power supply condition, and might supply a larger volume of PAM with a quite high level of RONS, which provides a significant advantage in large-area plasma treatments of biological samples. It was found that the APPJ array provides abundant reactive species to the liquid sample. The production of reactive species in the plasma-activated media exhibits a similar behavior to that in the gas phase. By varying the gas flow rate, pulse width of the applied voltage, and plasma exposure time, the composition of PAM can be modulated. Increasing gas flow rates led to higher concentration of H_2_O_2_ and NO_3_^-^. The RONS concentration were dependent on the media type, being higher in the order of DMEM, HBSS and DW. It was observed that the cell viability decreased with increasing RONS. Although the viability of PAM-treated cells exhibited a correlation with the RONS concentration in the PAM, the influence of pulse width on the viability of treated cells was not significant (especially for A549 cell). Finally, this study reveals that PAM-induced ROS can regulate the protein expression associated with mitochondria, and PAM causes apoptosis through Cyto C/JNK/p38 signaling on human cervical cancer cells. In a separate study [8] we reported that PAM inhibits tumor growth in a xenograft model with an increase in intracellular ROS and lipid ROS, thereby resulting in mitochondrial dysfunction without causing liver toxicity. This may suggest the feasibility of the PAM treatment in in-vivo condition. This work demonstrated that the APPJ array offer a compelling tool to deliver gaseous RONS into PAM for cancer cell treatment.

## Supporting information

S1 File Cell viability of various types of cancer cells treated by PAM. (PDF)
