# Supplementary material for: Plasma activated medium prepared by a bipolar microsecond-pulsed atmospheric pressure plasma jet array induces mitochondria-mediated apoptosis in human cervical cancer cells
Source: PLoS One. 2022 Aug 8;17(8):e0272805. doi: 10.1371/journal.pone.0272805 (PMC9359560; doi:10.1371/journal.pone.0272805)
Supplement: S1 File — (PDF) [file pone.0272805.s001.pdf]

Supporting Information

Plasma Activated Medium Prepared by a Bipolar Microsecond-Pulsed Atmospheric Pressure Plasma Jet Array Induces Mitochondria-mediated Apoptosis in Human Cervical Cancer Cell

A. Jo[a),1)], H. M. Joh[b),1)], J. H. Bae[b)], S. J. Kim[b)], T. H. Chung[b)]*, and J. W. Chung[a)]*

Comparison of cell viability in various types of cancer cells treated with PAM

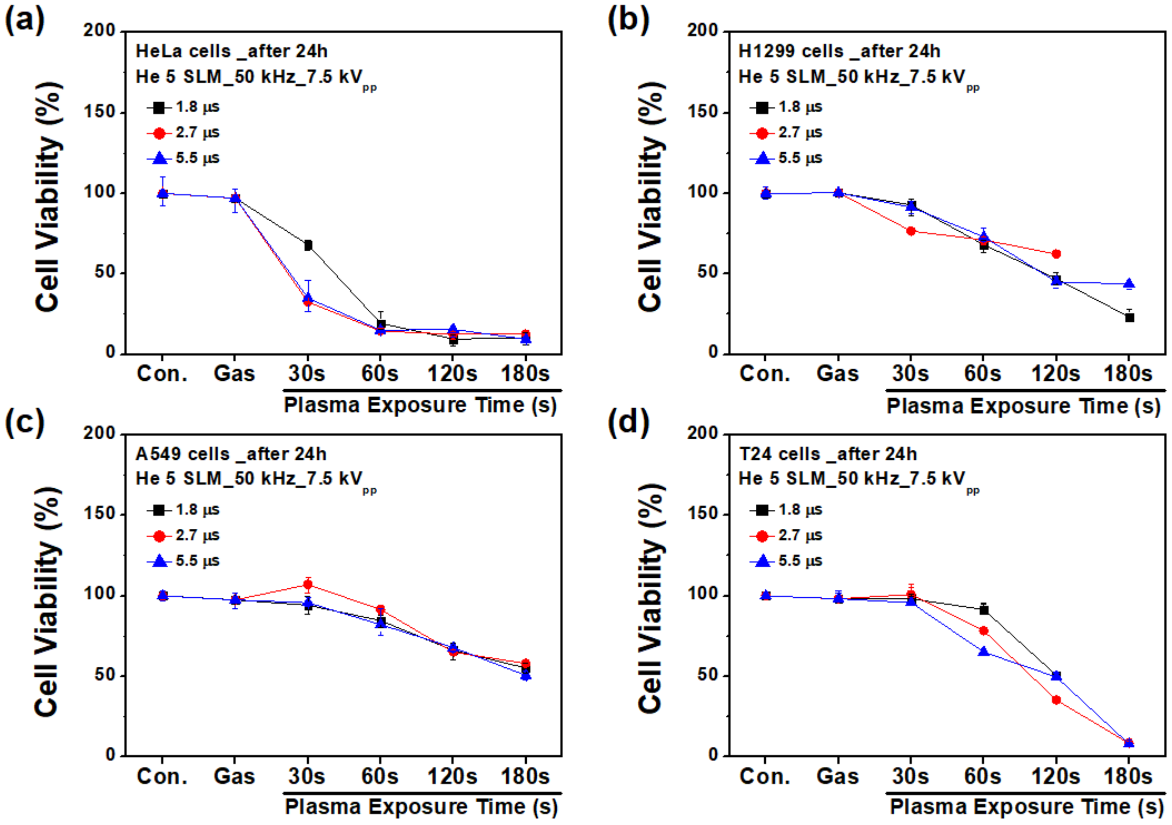

Fig S1. Cell viability in various types of cancer cells treated with PAM

We examined the ability of PAM to reduce cell viability in other types of cancer cells. Besides HeLa cell, human lung cancer cells (H1299 and A549) and human bladder cancer cells (T-24) were treated with PAM prepared at different pulse width (of applied voltage) and plasma exposure times. In order to confirm the viability of cancer cells, we used MTT solution (5 mg/mL). The cells were seeded in 96-well plates at a density of 1×10^4 cells/well. After the cells are attached, PAM was treated for 24 h. After incubation, PAM was removed and 100 μL MTT solution was added to each well and the cells were further incubated for 3 h. The violet crystals are solubilized with DMSO and the absorbance was measured at 550 nm using microplate reader (FLUO star OPTIMA, BMG Lab tech, Ortenberg, Baden-Wrttemberg, Germany). The relative cell viability (%) was calculated as (O.D. of

PAM-treated cells/O.D. of non-treated cells) × 100. PAM treatment resulted in a dose-dependent decrease in the viability. Although PAM reduced the viability of these cell lines with a similar dependence on the pulse width and plasma exposure times to that of HeLa cell, the influence of pulse width on the viability of A549 cells was not significant.
